# The mitogen-activated protein kinome from *Anopheles gambiae*: identification, phylogeny and functional characterization of the ERK, JNK and p38 MAP kinases

**DOI:** 10.1186/1471-2164-12-574

**Published:** 2011-11-23

**Authors:** Ashley A Horton, Bo Wang, Lauren Camp, Mark S Price, Arora Arshi, Mate Nagy, Steven A Nadler, James R Faeder, Shirley Luckhart

**Affiliations:** 1 Department of Medical Microbiology and Immunology, School of Medicine, 3146 Tupper Hall, One Shields Avenue, University of California, Davis, 95616, USA; 2 Department of Nematology, College of Agricultural and Environmental Sciences, 354 Hutchison Hall, One Shields Avenue, University of California, Davis, 95616, USA; 3 Department of Computational Biology, University of Pittsburgh School of Medicine. 3082 Biomedical Science Tower 3, 3501 Fifth Avenue, Pittsburgh, PA 15260, USA

## Abstract

**Background:**

*Anopheles gambiae *is the primary mosquito vector of human malaria parasites in sub-Saharan Africa. To date, three innate immune signaling pathways, including the nuclear factor (NF)-kappaB-dependent Toll and immune deficient (IMD) pathways and the Janus kinase/signal transducers and activators of transcription (Jak-STAT) pathway, have been extensively characterized in *An. gambiae*. However, in addition to NF-kappaB-dependent signaling, three mitogen-activated protein kinase (MAPK) pathways regulated by JNK, ERK and p38 MAPK are critical mediators of innate immunity in other invertebrates and in mammals. Our understanding of the roles of the MAPK signaling cascades in anopheline innate immunity is limited, so identification of the encoded complement of these proteins, their upstream activators, and phosphorylation profiles in response to relevant immune signals was warranted.

**Results:**

In this study, we present the orthologs and phylogeny of 17 *An. gambiae *MAPKs, two of which were previously unknown and two others that were incompletely annotated. We also provide detailed temporal activation profiles for ERK, JNK, and p38 MAPK in *An. gambiae *cells *in vitro *to immune signals that are relevant to malaria parasite infection (human insulin, human transforming growth factor-beta1, hydrogen peroxide) and to bacterial lipopolysaccharide. These activation profiles and possible upstream regulatory pathways are interpreted in light of known MAPK signaling cascades.

**Conclusions:**

The establishment of a MAPK "road map" based on the most advanced mosquito genome annotation can accelerate our understanding of host-pathogen interactions and broader physiology of *An. gambiae *and other mosquito species. Further, future efforts to develop predictive models of anopheline cell signaling responses, based on iterative construction and refinement of data-based and literature-based knowledge of the MAP kinase cascades and other networked pathways will facilitate identification of the "master signaling regulators" in biomedically important mosquito species.

## Background

Mitogen-activated protein kinases (MAPKs) are serine-threonine protein kinases that regulate a variety of cellular processes, including growth, metabolism, apoptosis, and innate immune responses [1-3]. MAPKs function in multi-tiered signaling cascades, in which an activated MAP4K phosphorylates and activates a MAP3K which, in turn, activates a downstream MAP2K, which activates a MAPK that can regulate effector proteins or transcription factors to positively or negatively regulate suites of genes [4,5]. MAPK signaling modules provide multiple levels of regulation that confer signal amplification and specificity toward a desired outcome [4]. A wide assortment of stimuli activate MAPKs, including inflammatory cytokines [6], osmotic stress [7], oxidative stress and redox signaling [8], and growth factors [9,10].

MAPKs have been extensively studied and a wealth of information is available from many model systems, including *Caenorhabditis elegans*, *Drosophila melanogaster *and a variety of mammals [11-13]. From an evolutionary standpoint, MAPKs have diverged very little over time and several published phylogenies of MAPKs have revealed a high degree of conservation from invertebrates to vertebrates [14,15]. Further, these analyses have contributed to our understanding of the evolution and function of the MAPKs [14,15]. For example, a MAPK phylogeny was constructed from the encoded sequences in the genome of the human pathogenic blood fluke, *Schistosoma japonicum*, together with known eukaryotic MAPKs from model organisms to elucidate putative functions of previously undescribed *S. japonicum *MAPKs [16]. The construction of MAPK phylogenies can, therefore, facilitate predictions of the roles of MAPKs in non-model organisms, including those of public health importance.

Malaria is a parasitic disease of great public health concern, with over 250 million new cases per year, resulting in nearly one million deaths annually [17]. In sub-Saharan Africa, the mosquito *Anopheles gambiae *transmits the most deadly human malaria parasite *Plasmodium falciparum*. Despite highly efficient transmission, the invertebrate and vertebrate hosts of malaria parasites can mount sophisticated immune responses to infection. These responses are regulated in both hosts, in part, by MAPKs [9,18-20].

Two prominent parasite-derived signals - glycosylphosphatidylinositols (GPIs) and hemozoin - activate MAPK signaling in both the mammalian and mosquito hosts. Mammalian JNK, ERK and p38 MAPKs transduce signals from *P. falciparum *glycophosphatidylinositols (PfGPIs) for inflammatory cytokine synthesis in immune cells *in vitro *and during parasite infection *in vivo *[18,19]. Hemozoin signals principally through ERK to increase interferon-gamma-dependent production of anti-parasite nitric oxide (NO) in mammalian cells [21,22]. In an analogous fashion, PfGPIs function as an early signal of parasite infection in *An. gambiae *[23] and in *Anopheles stephensi *[24], a vector of malaria in Asia and close relative of *An. gambiae*. In *An. stephensi*, PfGPIs robustly activate MEK-ERK phosphorylation in the mosquito midgut epithelium [24], a site that is critical for parasite development in the insect host. As in mammalian cells, hemozoin can activate MEK-ERK signaling in the *An. stephensi *midgut [25]. Further, transforming growth factor (TGF)-beta1-dependent MEK-ERK-dependent signaling can facilitate *P. falciparum *development at the midgut epithelium by inhibiting the expression of NO synthase [9] and synthesis of inflammatory levels of reactive nitrogen oxides that limit parasite development [26-28].

In contrast to our understanding of ERK signaling in *An. stephensi *and *An. gambiae*, our knowledge of the regulatory ligands and signaling pathways as well as the biological impacts of JNK and p38 MAPK signaling in these species is more limited. In particular, JNK activation appears to mediate as yet unidentified inhibitory responses to the murine parasite *Plasmodium berghei *in *An. gambiae *[29], although the extracellular signals and upstream regulatory proteins for JNK activation are unknown. In the case of p38 MAPK, this signaling protein appears to regulate antimicrobial responses in *Aedes *mosquitoes [30-32] and p38 MAPK is activated during insulin-dependent immune signaling in *An. stephensi *[20]. However, the pathway elements that regulate this signaling, the biological effects of signaling, and the identity of other activating ligands in *Anopheles *spp., including those derived from malaria parasites [25], are unknown.

In this study, we used bioinformatics, existing knowledge of mammalian signaling pathways, and signaling assays in *An. gambiae *cells to provide a more comprehensive understanding of the likely roles of the MAPK cascades in innate immunity of *An. gambiae*, a critical malaria vector mosquito for which an annotated genome sequence is available [33]. The MAPK phylogeny presented here recapitulates the conservation of the MAPKs and, for the first time, identifies the orthologous associations and evolutionary origins of the complete suite of *An. gambiae *MAPKs. The functional data presented herein demonstrate differing roles for *An. gambiae *p38 MAPK, ERK and JNK in response to stimuli that are biologically relevant for malaria parasite infection and, together with existing knowledge from mammalian models, suggest that anopheline mosquitoes utilize a unique MAPK architecture for signaling.

## Methods

### MAPK identification

The initial set of 43 human MAPKs used for reference in this study was identified in the GeneCards database [34] (Additional File 1). Fifteen *An*. *gambiae *MAPK orthologs were identified in GeneCards and confirmed using Basic Local Alignment Search Tool (BLAST) [35] against *An. gambiae *genome sequence data. In addition, *An. gambiae *sequence data were analyzed using highly conserved, orthologous MAPK activation loop sequences. The activation of MAP kinases occurs by a dual phosphorylation event by the upstream kinase on a solvent-exposed activation loop that typically contains a T-X-Y motif [36,37]. We used the encoded activation loop sequences from *D. melanogaster Slipper *(KTLKITDFGLAREAGTYAWMPPEVISV) and *D. melanogaster Wallenda *(EVVKISDFGTSREGTVAWMAPEVIRNPCSEKVDIWSY), flanked by 5-10 additional conserved amino residues, in TBLASTN queries against *An. gambiae *sequence data. Conserved catalytic domains for all predicted proteins were confirmed using Prosite.

### Phylogenetic analysis

For phylogenetic analyses, 108 sequences, ranging from 115-354 amino acids, of MAPK catalytic domains from *An. gambiae*, *C. elegans*, *Ciona intestinalis*, *D. melanogaster*, *Homo sapiens *and *Saccharomyces cerevisiae *were used to generate an alignment using the BLOSUM protein weight matrix and ClustalX [38]. Orthologs of *C. elegans*, *D. melanogaster *and *C. intestinalis *MAPKs were confirmed using BLASTP of GeneCards, Ensemble Metazoa, Wormbase and Flybase databases. Phylogenetic analyses were conducted using Phylogeny Inference Package (PHYLIP) 3.69 [39]. PROTDIST was used to generate a distance matrix based on the Jones-Taylor-Thornton (JTT) model of amino acid substitution. A distance-based phylogenetic tree was then inferred using the neighbor-joining (NJ) algorithm implemented in NEIGHBOR. SEQBOOT was used to prepare 1000 pseudoreplicate datasets for bootstrap analysis; these replicate datasets were then analyzed using PROTDIST with the JTT model and NEIGHBOR to create a bootstrap NJ tree. The bootstrap majority-rule consensus tree was generated using CONSENSE, visualized in FigTree v 1.3.1 http://tree.bio.ed.ac.uk/software/figtree/ and edited using Inkscape 0.48 http://inkscape.org/. The unrooted consensus tree was drawn to scale, with branch lengths representing the mean number of substitutions per site.

### Mosquito cell culture and experimental treatments

The immortalized, embryo-derived *An. gambiae *4a3B cell line (kindly provided by Hans-Michael Müller) [40] was maintained in Schneider's medium (Invitrogen, Carlsbad, CA) with 10% heat-inactivated fetal bovine serum (Invitrogen) at 28°C. For each condition, 1 × 10^6 ^4a3B cells in 2 mL medium were plated in one well of a 12-well tissue culture plate and allowed to recover overnight. Cells were then treated with the following stimuli at concentrations previously validated for signaling: 250 μM hydrogen peroxide (VWR International, Radnor, PA) [20], 6000 pg/ml human TGF-beta1 (R&D Systems, Minneapolis, MN) [20], 1.7 μM human insulin (Sigma-Aldrich, St. Louis, MO) [20] or 100 μg/ml lipopolysaccharide (LPS; Sigma-Aldrich) [41] and collected at 5 min, 15 min, 30 min, 1 h, 3 h, 6 h and 24 h after treatment for subsequent western blot analysis. To quantify and analyze ERK, JNK, and p38 MAPK transcript expression, 4a3B cells prepared as described above were treated with 1.7 μM human insulin and collected at 1 h, 3 h, 6 h and 24 h for qPCR (see below). Controls for each time point consisted of an identical aliquot of cells treated with diluent. At least three biological replicates were performed for each treatment.

### Protein extraction and western blotting

Protein extracts of 4a3B cells were prepared by collecting cells in lysis buffer as previously described [9,24]. Briefly, cell medium was removed following treatment and cells were washed with ice cold phosphate buffered saline (PBS) and lysed in the plate in 120 μl cell lysis buffer (10 mM Tris-HCl pH 7.4, 1 mM EDTA, 100 mM NaCl, 1 mM NaF, 1 mM EGTA, 2 mM Na_3_VO_4_, 20 mM Na_4_P_2_O_7_, 0.1% SDS, 1% Triton X-100, 0.5% sodium deoxycholate, 1 mM phenylmethylsulfonyl fluoride, 10% glycerol, 60 mg/ml aprotinin, 10 mg/ml leupeptin, 1 mg/ml pepstatin, and 1 mg/ml calyculin A). The plate was agitated for 30 min at 4°C and samples were incubated on ice for 30 min. Cell lysates were centrifuged at 14,000 × g for 10 min at 4°C to remove cellular debris and 100 μl of supernatant from each sample was mixed with 20 μl of 6 × sample buffer (125 mM Tris-HCl pH 6.8, 10% glycerol, 10% SDS, 0.006% bromophenol blue, 130 mM dithiothreitol) and heated at 95°C for 4 min.

Protein samples were electrophoretically separated on 10% polyacrylamide gels via sodium dodecyl sulfate polyacrylamide gel electrophoresis (SDS-PAGE) and transferred to nitrocellulose membranes (BioRad, Hercules, CA). Protein loading was visually assessed by Coomassie blue staining. Membranes were blocked in 5% nonfat dry milk (NFDM) in 1 × Tris-buffered saline containing 0.1% Tween-20 (TBS-T) for 1 h at room temperature. For phosphorylated ERK detection, membranes were incubated at 4°C overnight with 1:10,000 mouse anti-phospho-ERK monoclonal antibody (Sigma-Aldrich) in 5% NFDM in TBS-T. For detection of phospho-p38, phospho-JNK and the loading control protein glyceraldehyde 3-phosphate dehydrogenase (GAPDH), membranes were incubated at 4°C overnight with 1: 1,250 rabbit anti-phospho-p38 MAPK antibody (Cayman Chemical, Ann Arbor, MI), 1:1,250 rabbit anti-phospho-JNK1/2 antibody (Biosource, Carlsbad, CA), or with 1:10,000 rabbit anti-GAPDH antibody (Abcam, San Francisco, CA) in 5% NFDM in TBS-T. Membranes were washed 3 times for 5 min in 1 × TBS-T and incubated with a 1:20,000 dilution of horse radish peroxidase (HRP)-conjugated rabbit anti-mouse IgG (Sigma-Aldrich) or with a 1:20,000 dilution of HRP-conjugated goat anti-rabbit (Fab')2 fragment (Cell Signaling Technology, Danvers, MA) at 4°C overnight. Following incubation with the secondary antibody, membranes were washed 3 times for 5 min in 1 × TBS-T. To reveal antibody-bound proteins, membranes were incubated with SuperSignal West Pico chemiluminescent reagent (Pierce, Rockford, IL) for 2-3 min. Each membrane was exposed to blue autoradiography film (ISC Bioexpress, Kaysville, UT). Phospho-MAPK levels and total GAPDH levels were quantified on scanned film using a GS-800 calibrated densitometer (BioRad, Hercules, California).

### MAPK quantitative PCR (qPCR)

Primers for qPCR of *An. gambiae *ERK, p38, JNKa, and JNKb were designed using Primer3Plus http://www.bioinformatics.nl/cgi-bin/primer3plus/primer3plus.cgi. The primers included: ERK, 5'ATCCCGAGCACGATCACA3' (forward) and 5'CGATTTTGTGTAGCCCTTGGA3' (reverse); p38 MAPK, 5'CGGACCACATTCACCAGCTA3' (forward) and 5'CGCTAAAGTTGCGCTTCTCC3' (reverse); JNKa, 5'GCACGCAGCGATACATTAGC3' (forward) and 5'GTCCACCGAAATCCTTTCCA3' (reverse); and JNKb, 5'GACCGACTCGAACGAGCAC3' (forward) and 5'CGAACCACACGTTGATGTAGC3' (reverse). The housekeeping gene encoding ribosomal protein S7 was analyzed as a control with the primers 5'GAAGGCCTTCCAGAAGGTACAGA3' (forward) and 5'CATCGGTTTGGGCAGAATG3' (reverse).

RNA was isolated from 4a3B cells using Trizol regent (Invitrogen) and contaminating DNA was removed from the RNA samples using Turbo DNA-free™ (Ambion, Austin, TX). All RNA samples were diluted to 200 ng/μl and 6 μl (1.2 μg) RNA from each sample was reverse-transcribed using SuperScript^® ^III (Invitrogen). Sample cDNAs were used to perform qPCR using Maxima SYBR Green/ROX qPCR Master Mix (Fermentas, Glen Burnie, MD). The qPCR cycling conditions were as follows: 2 min at 50°C, 10 min at 95°C, and 40 cycles of 15 s at 95°C and 1 min at 60°C. Three biological replicates with independent batches of 4a3B cells were analyzed for the diluent controls and treatments. No template controls were included for each reaction plate and all reactions were performed in triplicate to confirm amplification consistency (e.g., all reactions within 0.5 Ct of each other). The triplicate data were used to determine an average Ct for each reaction and the data analyzed using the 2^-ΔΔCt ^method as described [9]. Briefly, the average Ct value for each target was first normalized to the average Ct for ribosomal protein S7 gene to obtain ΔCt, and then the ΔCt value for each insulin-treated sample was normalized to timepoint-matched control to obtain ΔΔCt. Fold-change of expression relative to the diluent control was determined using 2-ΔΔCt.

### Statistical analyses

For western blotting data, the Kolmogorov-Smirnov test revealed that non-normal distributions were present in our data sets (Graphpad Prism 5.02, San Diego, California). Overall significance values of all data sets, therefore, were determined by the Kruskal-Wallis test and Dunn's post test for pairwise comparisons (α = 0.05). For the q-PCR data, one way ANOVA and Student-Neuman-Keuls (Graphpad Prism 5.02) were used for analyses of significance (α = 0.05).

## Results

### MAPK discovery

We used a combination of bioinformatic techniques to identify a total of 17 MAPKs from the *An. gambiae *genome (Additional File 1). Among these, AGAP002710 and AGAP006461 were newly identified MAPK orthologs. Based on homology, AGAP002710 is most appropriately defined as *An. gambiae *MAP3K10/11 or mixed lineage kinase (MLK) 2/3 (Additional File 1). Similarly, AGAP006461 is most appropriately defined as *An. gambiae *MAP3K 12/13 (Additional File 1).

AGAP012148 and AGAP009460 are annotated as p38 MAPK and JNK orthologs, respectively, in Vectorbase [42]. However, inspection of AGAP012148 by alignment with p38 MAPK orthologs and Reverse Psi (RPS)-BLAST for conserved domains revealed that the annotated sequence was missing key N-terminal residues in the kinase catalytic domain. Specifically, RPS-BLAST of the annotated sequence predicted only 4 of 26 active site and 4 of 11 activation loop residues (not shown). Because the annotation appeared to be incomplete, we used the first 10 encoded amino acids from the N-terminus of the *Ae. aegypti *p38 MAPK ortholog (GenBank XP_001653239.1) to query the National Center for Biotechnology Information (NCBI) *An. gambiae *trace archives database (translated in all 6 reading frames) and identified the probable N-terminus of AGAP012148 (Additional File 2). Similarly, the annotation of AGAP009460 in Vectorbase [42] does not encode a start methionine. Approximately 150,000 base pairs of sequence upstream of the 5'-most nucleotides of AGAP009460 were translated in the three forward reading frames and analyzed by BLASTX using the first 20 amino acids of the corresponding orthologs from *Ae. aegypti *(AAEL008622) and from *Culex quinquefasciatus *(CPIJ001156, CPIJ001157). A putative start methionine with Kozak consensus and additional N-terminal amino acids extended the encoded sequence of AGAP009460 (Additional File 3).

The MAP4Ks, the MAP3Ks, the MAP2Ks are present in 1:1 orthology with gene products that have been identified in *D. melanogaster *(Additional File 1). Among the MAPKs, only *D. melanogaster *Rolled and *An. gambiae *ERK are in 1:1 orthology. In contrast, the *D. melanogaster *genome encodes two p38 MAPK orthologs (p38b, MPK2), a single JNK ortholog (Basket), an ERK5 ortholog (p38c), and an ERK7 ortholog, although the latter two are not associated with prototypical MAP kinase cascades [43]. The *An. gambiae *genome, on the other hand, encodes a single p38 MAPK, two JNK orthologs (JNKa, JNKb; 57% identical), and no ERK5 and ERK7 orthologs. Based on sequence homology, *An. gambiae *JNKb is most closely related to *D. melanogaster *Basket (Additional File 1). The lack of strict 1:1 orthology among the MAPKs is perhaps not surprising because diversification among these proteins to accommodate unique aspects of host biology would have a greater likelihood of being tolerated relative to upstream changes in the signaling cascades that could result in significantly amplified non-adaptive phenotypes.

To determine whether the complement of MAPKs in the *An. gambiae *genome was similar to that in other mosquito genomes, we examined the available ERK, JNK, and p38 MAPK orthologs for *Ae. aegypti *and *Cx. quinquefasciatus *(Additional File 4). The genome sequences of *Ae. aegypti *and *Cx. quinquefasciatus *are currently assembled into supercontigs in Vectorbase [42] (AaegL1.2, September 2009; CpipJ1.2, June 2008). In this context, if identical nucleotide sequences (due perhaps to sequence assembly) are excluded, ERK, JNKa, and JNKb orthologs are in 1:1:1 orthology among the three mosquito species. In contrast, *Cx. quinquefasciatus *appears to possess two relatively short MAPKs that are tentatively identified as p38 MAPKs. The encoded sequences share significant identity to *Ae. aegypti *p38 MAPK and the putative full-length *An. gambiae *p38 MAPK, but are non-overlapping sequences, so it is impossible to discern whether these are indeed truly distinct gene products encoded in the *Cx. quinquefasciatus *genome. Because these relationships cannot be confirmed with the existing genome assemblies, the *Ae. aegypti *and *Cx. quinquefasciatus *MAPK sequences were excluded from the phylogenetic analysis.

### Phylogeny

**Figure 1:**
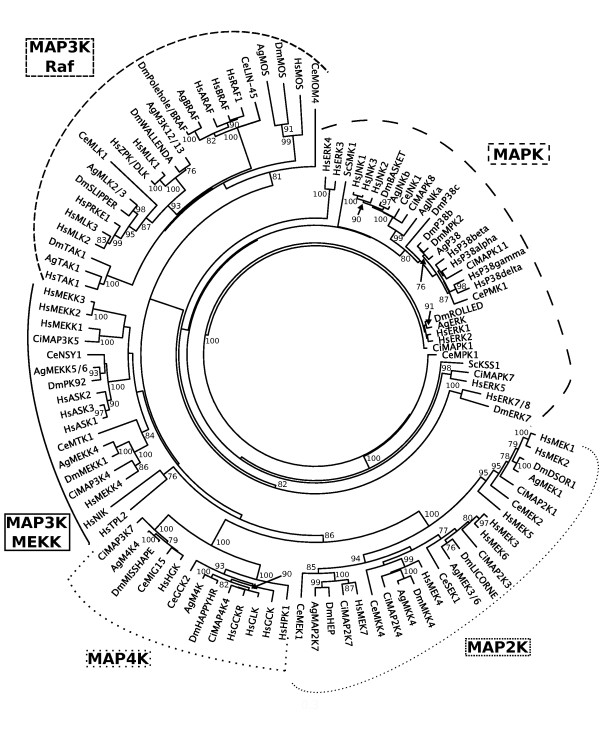
**Neighbor-joining (NJ) tree of MAPK catalytic domain sequences**. Unrooted NJ tree with results from bootstrap NJ analysis indicated for groups with support ≥ 75%. Groupings of the MAPK clades are marked. Abbreviations of species and NCBI accession numbers of MAPK sequences are as follows: *Anopheles gambiae *(AgERK XP_319983; AgJNKa XP_307879; AgJNKb XP_310236.3; AgM3K12/13 XP_316502.4; AgM4K XP_309868.4; AgM4K4 XP_316357.4; AgMAP2K7 XP_321199.4; AgMEK1 XP_322064.4; AgMEK3/6 XP_310813.4; AgMEKK4 XP_312585.4; AgMEKK5/6 XP_311281.4; AgMKK4 XP_314266.4; AgMLK2/3 XP_312218.4; AgMOS XP_308274.2; AgP38 XP_001689258.1; AgRAFPK XP_318144.4; AgTAK1 AGAP013516-PB (Ensembl ID, no current NCBI accession number)), *Caenorhabditis elegans *(CeLIN-45 AAR26307; CeGCK2 NP_504721; CeJNK1 NP_001021270; CeMEK1 NP_001024771; CeMEK2 NP_491087; CeMIG15 NP_001024971; CeMKK4 NP_509682; CeMLK1 NP_741537; CeMOM4 NP_492620; CeMPK1 NP_001022583; CeMTK1 NP_491683; CeNSY1 AAK31527; CePMK1 NP_501365; CeSEK1 NP_509322), *Ciona intestinalis *(CiMAP2K1 BAE06544.1; CiMAP2K3 BAE06545.1; CiMAP2K4 BAE06546.1; CiMAP2K7 BAE06548.1; CiMAP3K4 BAE06549.1; CiMAP3K5 XP_002122959.1; CiMAP3K7 (XP_002130898); CiMAP4K4 XP_002131009; CiMAPK1 BAE06412; CiMAPK7 BAE06414.1; CiMAPK8 BAE06525.1; CiMAPK11 BAE06625.1), *Drosophila melanogaster *(DmBASKET ACZ94221.1; DmDSOR1 AAF46475.1; DmERK7 AAF46481.2; DmHAPPYHR (happyhour) AAM70845.1; DmHEP (hemipterous) AAG22351.2; DmLICORNE AAF48223.1; DmMEKK1 AAF55592.2; DmMISSHAPE (misshapen) AAS64945.1; DmMKK4 ACZ94848.1; DmMOSPK NP_610817.1; DmMPK2 ACZ95005.1; DmP38b AAF53326.1; DmP38c AAS65203.1; DmPK92 AAF55711.3; DmPOLEHOLE/DmBRAF NP_001036258.1; DmROLLED EDP28108.1; DmSLIPPER AAF46344.3; DmTAK1 AAF50895.1; DmWALLENDA AAO41222.1), *Homo sapiens *(HsARAF NP_001645.1; HsASK1 NP_005914.1; HsASK2 NP_004663.3; HsASK3 NP_001001671.3; HsBRAF NP_004324.2; HsDLK/ZPK NP_006292.3; HsERK1 NP_002737.2; HsERK2 P28482.3; HsERK3 NP_002739.1; HsERK4 NP_002738.2; HsERK5 NP_002740.2; HsERK7/8 NP_620590.2; HsGCK NP_004570.2; HsGCKR Q9Y4K4.1; HsGLK NP_003609.2; HsHPK1 NP_009112.1; HsJNK1 NP_620637.1; HsJNK2 NP_002743.3; HsJNK3 NP_620448.1; HsMEK1 NP_002746.1; HsMEK2 NP_109587.1; HsMEK3 NP_659731.1; HsMEK4 NP_003001.1; HsMEK5 NP_660143.1; HsMEK6 NP_002749.2; HsMEK7 NP_660186.1; HsMEKK1 NP_005912.1; HsMEKK2 AAH_65755.1; HsMEKK3 NP_976226.1; HsMEKK4 Q9Y6R4.2; HsMLK1 NP_004712.1; HsMLK2 NP_002437.2; HsMLK3 NP_002410.1; HsMOS NP_005363.1; HsNIK NP_003945; HsHGK O95819.2; HsP38alpha NP_620581.1; HsP38beta NP_002742.3; HsP38delta NP_002745.1; HsP38gamma NP_002960.2; HsPRKE1 NP_149132.2; HsRAF1 P04049.1; HsTAK1 NP_663304.1; HsTPL2 NP_005195.2) and *Saccharomyces cerevisiae *(ScSMK1 856167; ScKSS1 AAZ22456.1). Aligned data and phylogenetic trees are available in TreeBASE http://www.treebase.org as study number S11970.

Phylogenetic analysis of the MAPK catalytic domains (Figure 1) showed strong support for the MAP4K and MAP2K groups (100% bootstrap support). The MAP3K Raf superfamily formed a group (81% bootstrap support), but the MAP3K MEKK family did not form an exclusive group. Similarly, the two MAP3K superfamilies did not group together as an exclusive group and the sequences representing MAPK were not supported as a group in this analysis. The lack of a close relationship between the Raf and MEKK MAP3K groups was not unexpected based on previous phylogenetic analyses [44,45]. The MEKK family proteins (MEKK1-4, ASK1-3) and the Raf superfamily proteins (MLK1-3, DLK, TAK1) are both associated with p38 and JNK signaling pathways; however, Raf proteins also figure prominently in MEK/ERK signaling (RAF, MOS) [43]. Although not reflected in this analysis, the previously noted division of the ERK subfamily (ERK1, 2, and 5 versus ERK3, 4, 7/8) may be due to the classification of ERK3, 4 and 7/8 as MAPK-like cascade independent kinases [43]. Frequently, sequences from the two deuterostomes (humans and *C. intestinalis*) and the insects (*An. gambiae *and *D. melanogaster*) grouped together. Exceptions to these groupings occurred when there were no clear homologs (e.g., HsMEK5 and HsPRKE1), which likely reflects the sequence diversification that has resulted in the many MAPKs that are found in humans relative to other species.

### Functional assays

The identification of the complement of MAP4Ks, MAP3Ks, MAP2Ks and MAPKs provides a framework for the prediction of patterns of activation of cell signaling cascades in *An. gambiae *cells. To attempt to determine whether these pathways are phenotypically represented in this mosquito species, we selected three stimuli (hydrogen peroxide, human insulin, and human TGF-beta1) that we had previously implicated in ERK- and p38 MAPK-dependent *An. gambiae *immune signaling [20], as well as lipopolysaccharide (LPS), an activator of immune signaling pathways in mosquito cells [41,46], to stimulate *An. gambiae *4a3B cells over an extended timecourse from 5 min to 24 h. A representative western blot is shown in Additional File 5.

**Figure 2:**
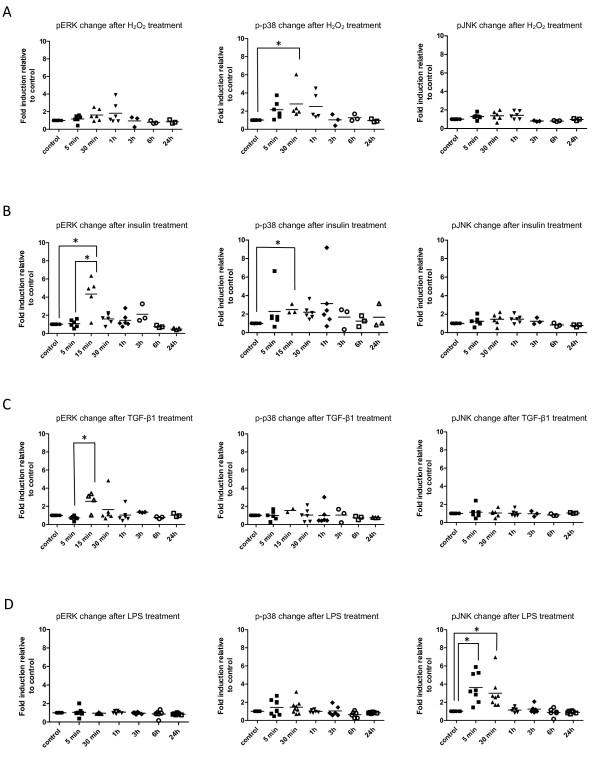
**Levels of pERK, p-p38 and pJNK after hydrogen peroxide (H_2_O_2_) (A), human insulin (B), human TGF-beta1 (C) or LPS (D) stimulation of *An. gambiae *4a3B cells**. Samples were collected at 5 min, 30 min, 1 h, 3 h, 6 h and 24 h after stimulation (n = 3-9 for each treatment and each timepoint). Levels of phospho-MAPKs were normalized to GAPDH and control levels and are indicated as fold change relative to control (indicated as "1"). For the sake of readability, a single control is indicated for each condition for each MAPK. Biological replicates of samples are shown as individual data points. Overall significance values of all data sets were determined by the Kruskal-Wallis test and Dunn's post test for pairwise comparisons (α = 0.05). * Denotes significant (p < 0.05) differences between treatments or between treatment and control groups connected by brackets.

Stimulation with 250 μM hydrogen peroxide induced significant activation of p38 MAPK at 30 min relative to the control group (Figure 2A, middle panel). Signaling declined from 30 min to 3 h post-stimulation, with a return to control levels by 3 h (Figure 2A, middle panel). Despite trends toward induction within 1 h of stimulation, ERK and JNK were not significantly activated by 250 μM hydrogen peroxide in 4a3B cells (Figure 2A, left and right panels). In previous studies, stimulation of 4a3B cells with 500 μM hydrogen peroxide induced activation of p38 MAPK as well as ERK by 15 min post-treatment [20], a faster and more extensive MAPK induction than observed here for 250 μM hydrogen peroxide (Figure 2A, middle panel).

In accord with previous observations at 15 min post-treatment [20], stimulation with 1.7 μM human insulin induced significant activation of ERK and p38 MAPK at 15 min relative to the control and 5 min time point (Figure 2B, left and middle panels). In contrast to the more temporally limited activation by hydrogen peroxide, insulin-activated ERK and p38 MAPK were detectable to 3 h and 24 h post-stimulation, respectively, suggesting a greater persistence of these signaling responses. Phosphorylation of JNK was slightly, although not significantly, elevated relative to control levels within 1 h after stimulation (Figure 2B, right panel).

Stimulation with 6000 pg/ml human TGF-beta1 induced significant activation of ERK only at 15 min post-treatment relative to the 5 min time point (Figure 2C, left panel), with detectable levels of activated ERK through 1 h post-stimulation. This induction of ERK activation at 15 min, together with a lack of p38 MAPK activation, was noted previously [20]. In contrast to slight trends toward JNK activation by hydrogen peroxide and insulin (Figures 2A and 2B, right panels), phosphorylated JNK levels never exceeded baseline control levels at all times after stimulation with human TGF-beta1 (Figure 2C, right panel).

Stimulation with 100 μg/ml LPS presented a unique pattern of MAPK activation in *An. gambiae *4a3B cells. In contrast to ERK and p38 MAPK activation by hydrogen peroxide, insulin and TGF-beta1 (Figures 2A-C), LPS stimulation did not induce mean phosphorylated ERK levels above baseline (Figure 2D, left panel) and only non-significant trends toward elevated phosphorylated p38 MAPK were noted through 30 min post-stimulation (Figure 2D, middle panel). Levels of phosphorylated JNK, however, were significantly increased by LPS stimulation (Figure 2D, right panel) at 5 min and 30 min post-treatment relative to control, with slightly elevated but not significant levels of phosphorylated JNK through 3 h post-treatment that declined to baseline control levels by 6 h post-treatment.

Although the majority of signaling protein genes do not exhibit transcriptional responsiveness to environmental stimuli (reviewed in [47]), we questioned whether we might detect a signaling response at the MAPK transcript level in *An. gambiae *cells. To address this question, we used qPCR to analyze expression levels of p38 MAPK, JNKa, JNKb, and ERK expression in response to human insulin, the only stimulus for which we detected changes in phosphorylation for more than one MAPK (Figure 2). In replicated assays, none of the four genes exhibited a significant change in expression from 1 h to 24 h after treatment with 1.7 μM human insulin (Additional File 6). Together with our phosphorylation data, these data suggest that MAPK responsiveness in *An. gambiae *cells occurs principally at the protein level.

## Discussion

**Figure 3:**
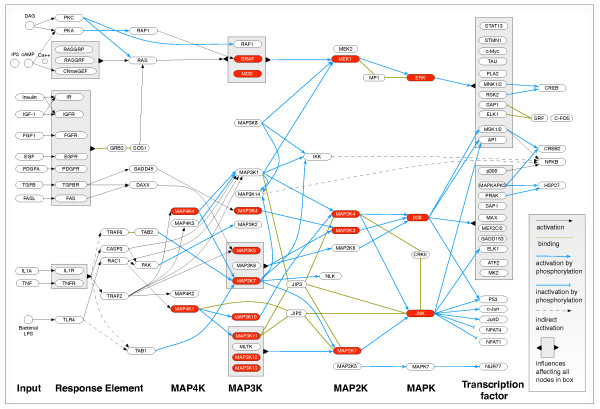
**An overview of mammalian MAPK signaling pathways**. This signaling diagram was adapted from the KEGG Pathway Database [48] updated with information from current literature [13,43]. OMNIGRAFFLE (The Omni Group, Seattle, WA) was used for drawing and layout. Signaling flows from left to right with ligands and receptors, followed by MAP4Ks, MAP3Ks, MAP2Ks and MAPKs. Orthologs identified in the genome of *An. gambiae *are shown in red. As shown in the legend on the right, arrows indicate directed interactions, e.g., phosphorylation, whereas lines indicate binding interactions. Indirect interactions are shown using dashed lines. Boxes are used to indicate sets of targets that share a common set of inputs or outputs.

The protein targets of the mammalian MAPKs (e.g., STAT, AP1, p300; Figure 3) [13,43,48] predict a strong association of the MAPKs with immune regulation. This association is also ancient, with strong representation in invertebrates. In *C. elegans*, p38 MAPK- and pERK-dependent signaling pathways, as well as insulin and TGF-beta signaling pathways, are required for host defense, with the general presumptions that insulin signaling functions in pathogen surveillance, p38 MAPK-dependent signaling functions in general defense, and p38 MAPK-independent signaling pathways provide pathogen-specific responses (reviewed in [49]). This nematode, however, lacks orthologs of Rel and nuclear factor (NF)-kappaB, the primary immune signaling transcription factors of *D. melanogaster*. In the fruit fly, Toll- or Imd-dependent signaling regulates NF-kappaB and JNK pathways, while p38 MAPK-dependent signaling mediates host defense against pathogen invasion [50,51]. The JNK and p38 MAPKs also mediate control of antimicrobial peptide expression in *D. melanogaster *[50,51], but cooperativity with NF-κB appears to be restricted to JNK. In particular, Imd activation results in the activation of TAK1 upstream of JNK and I-Kappa Kinase (IKK), the upstream activator of Rel that ultimately feeds back to reduce JNK activation [52].

The complement of MAP kinases encoded in the *An. gambiae *genome would provide this organism with the significant redundancy of p38 MAPK and JNK signaling observed in model organisms, although some intriguing differences between the mosquito and other species are predicted as well. In particular, *An. gambiae *MAP4K1 may function similarly to its mammalian counterpart downstream of Toll and RAC1 (AGAP005445) to activate the mosquito MLK2/3 ortholog (MAP3K10/11; Additional File 1) and p38 MAPK and JNK signaling (Figure 3). The second *An. gambiae *MAP4K - MAP4K4 - would be predicted to activate MAP3K1 and MAP3K7. However, *An. gambiae *lacks an ortholog for MAP3K1, an upstream activator for MEK/ERK signaling, so MAP4K4 activation of MAP3K7 (AgTAK1) would be predicted to activate NF-kappaB signaling through IKK (AGAP009166), and p38 MAPK and JNK signaling through MAP2K3 and MAP2K7 activation, respectively.

Among the other *An. gambiae *MAP3K orthologs, a number are predicted to lack well-defined upstream activators or to function independently of MAP4K activation. In particular, the activators of *An. gambiae *ortholog of MAP3K12/13 (Additional File 1) are not well defined based on data from mammalian models (Figure 3); nevertheless, the mosquito protein would be predicted to contribute to the activation of JNK signaling (Figure 3). Among the MAP3K orthologs that can be activated independently of MAP4Ks, *An. gambiae *MAP3K4 would be predicted to lie downstream of TGF-beta activation of GADD45 (AGAP007651), while MAP3K5 could be activated by TGF-beta-mediated activation of DAXX (AGAP009432) or TNF/TNFR activation of TRAF2 (Figure 3). Eiger and wengen have been identified as *D. melanogaster *orthologs of TNF/TNFR and, in the context of innate immunity, eiger functions independently of the Toll and Imd signaling pathways to regulate pathogen-induced cell death [53], proliferation of extracellular pathogens [54] and prophenoloxidase-mediated defense responses [55]. Although a clear ortholog of TRAF2 is not apparent in the *An. gambiae *genome, orthologs of eiger (AGAP006771) and wengen (AGAP000728) would suggest that signaling by this ligand and receptor could lead to MAP3K5/MAP2K3 activation of p38 MAPK.

An examination of possible pathways leading to MEK/ERK signaling based on the encoded MAP kinases reveals that, in addition to a lack of MAP3K1, *An. gambiae *also lacks an ortholog for MAP3K8, so the regulation of MEK/ERK signaling is likely dependent on B-RAF and MOS activation, which are the downstream targets for RAP1 (AGAP001874) and for RAS (AGAP002219, AGAP002812), a major transducer for multiple signaling inputs from growth factors and cytokines (e.g., insulin, IGF-1, and TGF-beta; Figure 3).

The data from our functional studies (Figure 2) not only confirm a subset of signaling predictions from known pathways (Figure 3), but also suggest that other pathways may be involved in *An. gambiae *MAPK activation. In particular, human insulin-induced activation of *An. gambiae *ERK was predicted (Figure 3) and this was consistent with previous observations [20]. In contrast, activation of *An. gambiae *p38 MAPK by insulin, which may occur through hydrogen peroxide at a concentration that is insufficient for ERK activation (Figure 2 compare panels A and B), is not predicted to flow through MAP4K-MAP3K-MAP2K activation (Figure 3). Interestingly, several studies have reported cross talk between the insulin receptor kinase and the Janus-activated kinase (JAK) signaling pathways [56-58]. In particular, JAK2 - the major mediator of inflammatory cytokine signaling in mammalian cells - can be activated by hydrogen peroxide [59] and contributes to insulin-dependent activation of p38 MAPK, JNK, and ERK in a manner that is independent of insulin receptor substrate activation and activation of the phosphatidylinositol 3-kinase/Akt-dependent arm of insulin signaling [60]. Jaramillo-Gutierrez *et al*. [29] proposed, based on the regulation of antioxidant gene expression by JNK, that this MAPK may in fact be induced by oxidative stress to control redox biology in *An. gambiae*. These observations - together with our data and known models of MAPK signaling (Figure 3) - predict the existence of a pathway involving insulin-induced hydrogen peroxide regulation of a JAK ortholog (AGAP008354) and downstream activation of p38 MAPK and JNK that can be tested experimentally in *An. gambiae*.

In contrast to the prediction of ERK-specific signaling by insulin, existing mammalian signaling data (Figure 3) predict that human TGF-beta1 should activate ERK as well as p38 MAPK in *An. gambiae *cells. We have extensively characterized TGF-beta1-dependent ERK signaling in the regulation of *P. falciparum *development in *An. stephensi *[9,20] and we have also demonstrated that p38 MAPK is activated by TGF-beta1 in *An. stephensi *cells [20]. Given that p38 MAPK can be activated in *An. gambiae *cells (Figure 2B, center panel), our understanding of mammalian MAPK signaling (Figure 3) would suggest that perhaps a deficiency in the TGFBR-GADD45-MAP3K4 cascade is responsible for the failure of TGF-beta1 to activate p38 MAPK in *An. gambiae *4a3B cells. A comparative analysis of *An. stephensi *and *An. gambiae *4a3B cells, therefore, could be used to identify critical differences in these signaling pathways.

LPS has been used as an activator of a variety of immune signaling pathways in mosquito cells [41,46]. A putative *An. gambiae *RAC1-MAP4K1-MAP3K11-MAP2K7-JNK signaling cascade (Figure 3) could provide one mechanism for LPS activation of JNK in *An. gambiae *4a3B cells (Figure 2D, right panel). However, we could also predict, given that this mosquito MAP3K is orthologous with MAP3K10 as well as MAP3K11 that LPS should also activate p38 MAPK signaling through MAP2K4. Because LPS does not significantly activate p38 MAPK in *An. gambiae *4a3B cells (Figure 2D, middle panel), we could speculate that this *An. gambiae *MAP3K is functionally more analogous to MAP3K11. An examination of MAP2K4 and MAP2K3 activation downstream of *An. gambiae *MAP3K10/11 using inhibitors or knock down strategies could clarify the orthology and functionality of this mosquito MAP3K.

Although LPS was the only pathogen-associated molecular pattern (PAMP) used in these signaling studies, the restriction of MAPK activation by LPS to *An. gambiae *JNK is insightful. Akman-Anderson *et al*. [25] showed that *P. falciparum *hemozoin induced activation of TAK1 and ERK as well as the signaling kinases Akt/protein kinase B and atypical protein kinase C zeta/lambda in *An. gambiae *4a3B cells. Activation of TAK1 by this important parasite signaling factor, together with LPS-specific activation of JNK in our studies, suggests that JNK may function specifically in pathogen detection in *An. gambiae *cells. Indeed, following infection with *P. berghei *or *P. falciparum*, the mosquito midgut epithelium undergoes profound cytoskeletal changes similar to those regulated by the Toll/MAP2K4/JNK signaling module in *D. melanogaster *[61,62].

Additional support of the importance, and perhaps specificity, of JNK signaling for control of natural *P. falciparum *infection in *An. gambiae *is provided by two population genetics studies. We identified a single nucleotide polymorphism (SNP) in the *An. gambiae *MAP2K4 (MKK4) gene that was in linkage disequilibrium with a SNP in the gene encoding Toll5B that was significantly associated with *P. falciparum *infection [63]. This synonymous SNP, MKK43, introduces a reduction of codon frequency greater than 3-fold, which could result in changes in downstream protein expression or function and, hence, JNK signaling [63]. In addition to our work, Riehle *et al*. [64] mapped a locus that is strongly associated with *An. gambiae *resistance to *P. falciparum *infection in east Africa that encompasses the chromosomal locations of MAP4K4 and MAP3K12, both of which are predicted to regulate JNK signaling (Figure 3).

## Conclusions

In sum, we have identified the complement of *An. gambiae *MAP kinases and used a combination of predicted signaling relationships and bioassays to suggest novel interactions and functionality that can be tested experimentally in this biomedically important mosquito species. Clearly, the MAPK signaling cascades are the major regulators of innate immunity in *C. elegans*, in *D. melanogaster*, and in mammals, but we are only beginning to appreciate the importance of these complex cascades in innate immunity of the mosquito genera that serve as vectors of globally devastating pathogens of humans. The establishment of this "road map" based on the most advanced mosquito genome annotation can, therefore, accelerate our understanding of host-pathogen interactions and broader physiology in *An. gambiae *and in the closely related *An. stephensi *and provide a reasonable architecture for similar efforts in *Ae. aegypti *and *Cx. quinquefasciatus*. Further, future efforts to develop predictive models of anopheline cell signaling responses, based on iterative construction and refinement of data-based and literature-based knowledge of the MAP kinase cascades and other networked pathways (reviewed in [65]), will ultimately be necessary to elucidate the "master signaling regulators" in these biomedically important insects.

## List of abbreviations

ASK: Apoptosis Signal-Regulating Kinase; BLASTX: Basic Local Alignment Search Tool for translated query against a protein database (X); DAXX: Death-associated protein 6; DLK: Dual Leucine zipper Kinase; ERK: Extracellular signal-Regulated Kinase; GADD45: Growth Arrest and DNA-Damage inducible protein; GAPDH: Glyceraldehyde 3-Phosphate Dehydrogenase; HRP: Horse radish peroxidase; IKK: I Kappa b Kinase; JAK-STAT: Janus Kinase/Signal Transducers and Activators of Transcription; JNK: Jun Kinase; LPS: Lipopolysaccharide; MAP4K: Mitogen-Activated Protein Kinase Kinase Kinase Kinase; MAP3K: Mitogen-Activated Protein Kinase Kinase Kinase; MAP2K: Mitogen-Activated Protein Kinase Kinase; MAPK: Mitogen-activated Protein Kinase; MEK: Equivalent to MAP2K; MEKK: Equivalent to MAP3K; MLK: Mixed Lineage Kinase; NFDM: Nonfat Dry Milk; NF-kappaB: Nuclear Factor-kappaB; NO: Nitric Oxide; PfGPIs: *Plasmodium falciparum *glycosylphosphatidylinositols; PRKE1: Equivalent to MAP3K9, MLK1; RAC1: Ras-related C3 botulinum toxin substrate 1; RAP1: Ras-related Protein 1; TAK1: TGF-beta-activated Kinase 1; TBLASTN: Basic Local Alignment Search Tool for protein query against a translated database (T); TBS-T: 1 × Tris-Buffered Saline with 0.1% Tween 20; TGF-beta1: Transforming Growth Factor-beta1; TNFR: Tumor Necrosis Factor Receptor; TRAF2:/6: TNFR-associated Factor 2/6.

## Authors' contributions

AAH and MSP confirmed the identities of the annotated and unannotated *An. gambiae *MAPKs, co-wrote the initial draft, and assisted in the preparation of figures/files. AAH also edited the manuscript and revisions. BW performed the MAPK signaling and expression assays with *An. gambiae *cells, analyzed the data, and edited the manuscript and revisions. LC and SAN performed the phylogenetic analyses, prepared and edited the phylogram, and edited the manuscript. AA, MN and JRF created the MAPK signaling diagram based on the KEGG Pathway Database and current literature and edited the manuscript. SL conceived of this work, directed the studies, and co-wrote the initial and final drafts for publication. All authors read and approved the final manuscript.

## Supplementary Material

Additional file 1 **MAPKs used in this study**. *H. sapiens*, *D. melanogaster*, *C. elegans *and *C. intestinalis *are listed by common names and by accession number. *An. gambiae *MAPKs are listed by Vectorbase AGAP numbers as well as by NCBI accession numbers and common names. Dashes indicate the absence of identified orthologs. N/A for *An. gambiae *MAP3K7 indicates no available NCBI accession number. Click here for file

Additional file 2 **Alignment of predicted mosquito p38 MAPKs**. Alignment of *An. gambiae *(AGAP), *Ae. aegypti *(AAEL) and *Cx. quinquefasciatus *(CPIJ) p38 MAPKs. The *Ae. aegypti *and *Cx. quinquefasciatus *sequences were used to query the *An. gambiae *trace archives database to identify the probable N-terminal amino acids of *An. gambiae *p38 MAPK (bold, underlined). Click here for file

Additional file 3 **Alignment of *An. gambiae *JNKa and *Ae. aegypti *and *Cx. quinquefasciatus *JNK orthologs**. Alignment of the first 20 amino acids of *Ae. aegypti *(AAEL) and *Cx. quinquefasciatus *(CPIJ) JNK orthologs with upstream sequence from AGAP009460 was used to predict the start methionine and additional N-terminal amino acids (bold, underlined) of *An. gambiae *JNKa. Click here for file

Additional file 4 **Predicted amino acid sequences of ERK, JNK, p38 MAPK orthologs from *Ae. aegypti *and *Cx. quinquefasciatus***. *Ae. aegypti *and *Cx. quinquefasciatus *MAPK orthologs as predicted from the supercontig assemblies for genome sequences for these species. These sequences were excluded from the phylogeny and, therefore, the relationships of these orthologs with *An. gambiae *MAPKs cannot definitively be confirmed. Click here for file

Additional file 5 **LPS-induced MAPK phosphorylation in *An. gambiae *4a3B cells**. Cells were treated with 100 μg/ml LPS or an equivalent volume of PBS. Cells were collected at 5 min, 30 min, 1 h, 3 h, 6 h and 24 h after treatment. MAPK phosphorylation was examined by western blotting as described in the Methods. GAPDH levels provided an assessment of protein loading and were used to normalize corresponding phospho-MAPK levels. This figure is a representative of immunoblots from 6-9 independent experiments. Click here for file

Additional file 6 **MAPK transcript expression in insulin-stimulated *An. gambiae *4a3B cells**. Cells were treated with 1.7 μM human insulin or an equivalent volume of diluent and collected at 1 h, 3 h, 6 h and 24 h after treatment (n = 3 for control and treatment at each timepoint). Expression levels of ERK (A), p38 MAPK (B), JNKa (C), and JNKb (D) were analyzed by qPCR as described in the Methods. Expression of ribosomal protein S7, a housekeeping gene control, was used for normalization of treatment and control expression. Insulin-treated MAPK expression levels are shown as fold changes relative to the timepoint-matched control group levels. Click here for file
