# Supplementary material for: The mitogen-activated protein kinome from Anopheles gambiae: identification, phylogeny and functional characterization of the ERK, JNK and p38 MAP kinases
Source: BMC Genomics. 2011 Nov 23;12:574. doi: 10.1186/1471-2164-12-574 (PMC3233564; doi:10.1186/1471-2164-12-574)
Supplement: Additional file 2 — Alignment of predicted mosquito p38 MAPKs. Alignment of An. gambiae (AGAP), Ae. aegypti (AAEL) and Cx. quinquefasciatus (CPIJ) p38 MAPKs. The Ae. aegypti and Cx. quinquefasciatus sequences were used to query the An. gambiae trace archives database to identify the probable N-terminal amino acids of An. gambiae p38 MAPK (bold, underlined). [file 1471-2164-12-574-S2.PDF]

```
AGAP012148       1   MPKFYRTEIN KTEWEVPEKY QVLTPVGSGA YGQVCSAMDT EHNVKVAIKK
AAEL008379       1   MPQFYKVEIN KTEWEVPQKY QMLTPVGSGA YGQVCSATDT QHNVKVAIKK
CPIJ002174       1   mvapgralvt n--------- ---------- -----SATDS QHNAKVAIKK
CPIJ002175       1   ri-------- ---------- ---------- ---------- ----------
                     ********** ********** ********** ********** **********
                     ********** ********** ********** ********** **********
                                                           ** ** **********

AGAP012148      51   LARPFQSAVH AKRTYRELRM LKHMNHENII GLLDVFHPGa nNTLESFQQV
AAEL008379      51   LARPFQSAVH AKRTYRELRM LKHMNHENII GLLDVFHPGG -NTLDSFQQV
CPIJ002174      27   LARPFQSAVH AKRTYRELRM LKHMNHENII GLLDVFHPGG -NTLDSFQQV
CPIJ002175       3   ---------- ---------- ---------- ---------- ----------
                     ********** ********** ********** **********  *********
                     ********** ********** ********** **********  *********
                     ********** ********** ********** *********   *********

AGAP012148     101   YLVTHLMGAD LNNIIRTQRL SDEHVQFLVY QILRGLKYIH SAGIIHRDLK
AAEL008379     100   YLVTHLMGAD LNNIIRTQRL SDDHVQFLVY QILRGLKYIH SAGIIHRDLK
CPIJ002174      76   YMVTHLMGAD LNNIIRTQRL SDDHVQFLVY QILRGLKYIH SAGIIHRDLK
CPIJ002175       3   ---------- ---------- ---------- ---------- ----------
                     ********** ********** ********** ********** **********
                     ********** ********** ********** ********** **********
                     ********** ********** ********** ********** **********

AGAP012148     151   PSNIAVNEDC ELKILDFGLA RPTENEMTGY VATRWYRAPE IMLNWMHYNQ
AAEL008379     150   PSNIAVNEDC ELKILDFGLA RPTENEMTGY VATRWYRAPE IMLNWMHYNQ
CPIJ002174     126   PSNIAVNEDC ELKILDFGLA RPTENEMTGY VATRWYRAPE IMLNWMHYNQ
CPIJ002175       3   ---------- ---------- ---------- ---------- ----------
                     ********** ********** ********** ********** **********
                     ********** ********** ********** ********** **********
                     ********** ********** ********** ********** **********

AGAP012148     201   TVDIWSVGCI MAELLTGRTL FPGTDHIHQL NLIMeilGTP NDEFMAKISS
AAEL008379     200   TVDIWSVGCI MAELLTGRTL FPGTDHIDHL TRIMffcGTP DDELMQKITS
CPIJ002174     176   TVDIWSVGCI MAELLTSRTL FPGTDp---- ---------- ----------
CPIJ002175       3   ---------- ---------- ---------- ---------- ----------
                     ********** ********** ********** ****   *** **********
                     ********** ********** *****             *** **********
                     ********** ********** *****

AGAP012148     251   ESARHYIKSL PKTEKRNFSD VFRGANPLAI DLLEKMLELD ADKRITAEQA
AAEL008379     250   EEARHYIRSL PKTEKRNFSD VFRGANPLAI DLLEKMLELD ADKRVTAEQA
CPIJ002174     202   ---------- ---------- ---------- ---------- ----------
CPIJ002175       3   ---------- ---------- ---------- ---------- -----TAEQA
                     ********** ********** ********** ********** **********
                     ********** ********** ********** ********** **********
                                                                      *****

AGAP012148     301   LAHPYLEKYA DPSDEPTSSL YDQSFEDMDL PVERWKELVF KEVLNFVPQQ
AAEL008379     300   LAHPYLEKYA DPTDEPTSSL YDQSFEDMDL PVEKWKELVF QEVLNFVPQQ
CPIJ002174     202   ---------- ---------- --------DL SITE------ ----------
CPIJ002175       8   LAHPYLEKYA DPTDEPTSSL YDQSFEDMDL PVEKW----- KEVVNFVPQQ
                     ********** ********** ********** ********** **********
                     ********** ********** ********** ********** **********
                     ********** ********** ********** *****      **********

AGAP012148     351   HAHIGGEPQa
AAEL008379     350   HAHIGSDGQ-
CPIJ002174     208   ----------
CPIJ002175      53   HAHIGGDAQ-
                     *********
                     *********
                     *****   *
```
