# Supplementary material for: The mitogen-activated protein kinome from Anopheles gambiae: identification, phylogeny and functional characterization of the ERK, JNK and p38 MAP kinases
Source: BMC Genomics. 2011 Nov 23;12:574. doi: 10.1186/1471-2164-12-574 (PMC3233564; doi:10.1186/1471-2164-12-574)
Supplement: Additional file 3 — Alignment of An. gambiae JNKa and Ae. aegypti and Cx. quinquefasciatus JNK orthologs. Alignment of the first 20 amino acids of Ae. aegypti (AAEL) and Cx. quinquefasciatus (CPIJ) JNK orthologs with upstream sequence from AGAP009460 was used to predict the start methionine and additional N-terminal amino acids (bold, underlined) of An. gambiae JNKa. [file 1471-2164-12-574-S3.PDF]

```
CPIJ001156      MAY-ANFPLPELLNEINNN-------------ASNTEFTVPGRFQN--LFPIGIGAQGAV 44
CPIJ001157      MAY-ANFPLPELLNEINNN-------------ASNTEFTVPGRFQN--LFPIGIGAQGAV 44
AAEL008622      MADGGNLNNVEVPYAMNHQPYA---------IEPQVYFMVPDRFEL--AHQLGIGAQGAV 49
AGAP009460      MQS--QEEAVEGTEGVHNKPAGGDPAPANPPSNEFYFHLPARFPASHMMPIGIGVQGAV 58
                *    :    *    ::::               :  * :* **       :***.****

CPIJ001156      CAAMDVVTGRPVAIKKLSRPFQDVTHAKRAYREIKLMRLVDHPFIIKLLHAYSPQNSLDT 104
CPIJ001157      CAAMDVVTGRPVAIKKLSRPFQDVTHAKRAYREIKLMRLVDHPFIIKLLHAYSPQNSLDT 104
AAEL008622      VAAIDRTNGNKVAVKKLSRPLENQTNAKRAYREIKLLQTLDHPFIIKLLYAYSPQNDLAS 109
AGAP009460      CSAVDIKTGRRLAVKKLSQPFQDVTFAKRAYRELKLMRLVDHPNIIKLLYAYTPQQTLDT 118
                 :*:*  .*. :*:****:*::: * *******:**:: :*** *****:**:**: * :

CPIJ001156      FRDIYLFTERMDTNLSVVIGNPLDHERLSFLVYQMLCGVKYLHSAGIIHRDLKPTNIVVR 164
CPIJ001157      FRDIYLFTERMDTNLSVVIGNPLDHERLSFLVYQMLCGVKYLHSAGIIHRDLKPTNIVVR 164
AAEL008622      FRDIYLFTECMDGNLSTVVGSPLDHERISFLIYQILCGIKHLHSAGIIHRDLKPTNIVVN 169
AGAP009460      FRDVYIFTELMDSSLQHVFGTKLDHERISFLVYQMLCGIRYLHSAGIIHRDLKPSNIVVR 178
                ***:*:*** **  .*.  *.*.  *****:***:**:***:.:************:****.

CPIJ001156      ADCSLKILDFGLAKVVGTNFMMTQYVVTRYYRAPEVILNMEYDTKVDIWAIGCIMAELIT 224
CPIJ001157      ADCSLKILDFGLAKVVGTNFMMTQYVVTRYYRAPEVILNMEYDTKVDIWAIGCIMAELIT 224
AAEL008622      KDCSLKILDFGLARSVGTNFNMTQYVITRYYRAPEVILNMDYDTNVDIWAIGCIMAELIK 229
AGAP009460      KNCTLKILDFGLARSIDTSFTMTQYVVTRHYRAPEIILNMEYDTKVDLWSIGCIMAELIT 238
                 :*:*********: :.*.* *****:**:*****:****:***:**:*:*********.

CPIJ001156      GRVLFPGTDHVDQWNKIVETLGTPTPELIAKAPSSERRYIETLPVHPRPTIEQLFPDESF 284
CPIJ001157      GRVLFPGTDHVDQWNKIVETLGTPTPELIAKAPSSERRYIETLPVHPRPTIEQLFPDESF 284
AAEL008622      GQVLLPGTDHVDQWNQITATLGTPSPEFMARASASTRNYIQKLPITPRPSFDVLFPDSDF 289
AGAP009460      GAVLFPGTDHVDQWMRIVETLGTPRPEFIARTTPGTQRYISNQPVVAGRPFGELFPDEVF 298
                * **:********* :*. ***** **::*::... :.**.. *: .  .:  ****. *

CPIJ001156      LATAA-GSPELNNANARAMLARMLTIDPAERMSTEEALAHPYISLWFQEDEVNRQAPVPY 343
CPIJ001157      LATAA-GSPELNNANARAMLARMLTIDPAERMSTEEALAHPYISLWFQEDEVNRQAPVPY 343
AAEL008622      LEEKN-DHSEVNNRNARDMLDRMLTIDPLNRMTVEEALTHPYVRCWLDEAEVNRPAPVPY 348
AGAP009460      EGMASPNVPQLTNDAARDFLRRMLAFDPMERISVDEALAHPYVSVWYYEEDVMRPPPKPY 358
                      . .::.*  ** :* ***::** :*::.:***:***:  *  * :* * .* **

CPIJ001156      DHALDEQELSLDQWKALLFQEIREIQAETLEAE-------------------------- 376
CPIJ001157      DHALDEQELSLDQWKALLFQEIREIQAETLEAEAVESESESEPESVESGLFGDLESESES 403
AAEL008622      DHTLDEQELSLDQWKALLFRDVKEIQAQTLG---------------------------- 379
AGAP009460      DHALDARNLTVEQWKRLLFEEIQDIQRTMSLDEA------------------------- 392
                **:** ::*::*** ***.::::**

CPIJ001156      -----------------
CPIJ001157      ESSKLEQLESESEPESA 420
AAEL008622      -----------------
AGAP009460      -----------------
```
