# Supplementary material for: The mitogen-activated protein kinome from Anopheles gambiae: identification, phylogeny and functional characterization of the ERK, JNK and p38 MAP kinases
Source: BMC Genomics. 2011 Nov 23;12:574. doi: 10.1186/1471-2164-12-574 (PMC3233564; doi:10.1186/1471-2164-12-574)
Supplement: Additional file 4 — Predicted amino acid sequences of ERK, JNK, p38 MAPK orthologs from Ae. aegypti and Cx. quinquefasciatus. Ae. aegypti and Cx. quinquefasciatus MAPK orthologs as predicted from the supercontig assemblies for genome sequences for these species. These sequences were excluded from the phylogeny and, therefore, the relationships of these orthologs with An. gambiae MAPKs cannot definitively be confirmed. [file 1471-2164-12-574-S4.PDF]

Orthologous *Ae. aegypti* and *Cx. quinquefasciatus* sequences for AGAP009207

Ae. aegypti ERK1/2 AAEL013939
MLWVDLGMRSAVDTVTKTKVAIKKISPFEHQTYCQRTLREIKILTRFKHENIIDIRDILR
VPSIEQMKDVYIVQCLMETDLYKLLKTQRLSNDHICYFLYQILRGLKYIHSANVLHRDLK
PSNLLLNTTCDLKICDFGLARVADPEHDHTGFLTEYVATRWYRAPEIMLNSKGYTKSIDI
WSVGCILAEMLSNRPIFPGKHYLDQLNHILGVLGSPSQEDLECIINEKARSYLQSLPYKP
KVPWSRLFPNADSNALDLLGKMLTFNPHNRISVEEALAHPYLEQYYDPADEPVAEEPFRI
AMELDDLPKETLKRLIFEETLRFNHNDNHPDVM

Ae. aegypti ERK1/2 AAEL007958
MKDVYIVQCLMETDLYKLLKTQRLSNDHICYFLYQILRGLKYIHSANVLHRDLKPSNLLL
NTTCDLKICDFGLARVADPEHDHTGFLTEYVATRWYRAPEIMLNSKGYTKSIDIWSVGCI
LAEMLSNRPIFPGKHYLDQLNHILGVLGSPSQEDLECIINEKARSYLQSLPYKPKVPWSR
LFPNADSNALDLLGKMLTFNPHNRISVEEALAHPYLEQYYDPADEVIVLNFALAWERNGG
YVDDM

Cx. quinquefasciatus ERK1/2 CPIJ005303
MLLFSTIYEWHANGVSLIIDIRDILRVPSIEQMKDVYIVQCLMETDLYKLLKTQRLSNDH
ICYFLYQILRGLKYIHSANVLHRDLKPSNLLLNTTCDLKICDFGLARVADPEHDHTGFLT
EYVATRWYRAPEIMLNSKGYTKSIDIWSVGCILAEMLSNRPIFPGKHYLDQLNHILGVLG
SPSQEDLECIINEKARSYLQSLPYKPKVPWSRLFANADPNALDLLGKMLTFNPHNRISVE
EALAHPYLEQYYDPADEPVAEEPFRIAMELDDLPKETLKRLIFEETLRFNHNDNLPDSM

Orthologous *Ae. aegypti* and *Cx. quinquefasciatus* sequences for AGAP009460

Ae. aegypti JNK AAEL008622
MADGGNLNNVEVPYAMNHQPYAIEPQVYFMVPDRFELAHQLGIGAQGAVVAAIDRTNGNK
VAVKKLSRPLENQTNAKRAYREIKLLQTLDHPFIIKLLYAYSPQNDLASFRDIYLFTECM
DGNLSTVVGSPLDHERISFLIYQILCGIKHLHSAGIIHRDLKPTNIVVNKDCSLKILDFG
LARSVGTNFNMTQYVITRYYRAPEVILNMDYDTNVDIWAIGCIMAELIKGQVLLPGTDHV
DQWNQITATLGTPSPEFMARASASTRNYIQKLPITPRPSFDVLFPDSDFLEEKNDHSEVN
NRNARDMLDRMLTIDPLNRMTVEEALTHPYVRCWLDEAEVNRPAPVPYDHTLDEQELSLD
QWKALLFRDVKEIQAQTLG

Cx. quinquefasciatus JNK CPIJ001156
MAYANFPLPELLNEINNNASNTEFTVPGRFQNLFPIGIGAQGAVCAAMDVVTGRPVAIKK
LSRPFQDVTHAKRAYREIKLMRLVDHPFIIKLLHAYSPQNSLDTFRDIYLFTERMDTNLS
VVIGNPLDHERLSFLVYQMLCGVKYLHSAGIIHRDLKPTNIVVRADCSLKILDFGLAKVV
GTNFMMTQYVVTRYYRAPEVILNMEYDTKVDIWAIGCIMAELITGRVLFPGTDHVDQWNK
IVETLGTPTPELIAKAPSSERRYIETLPVHPRPTIEQLFPDESFLATAAGSPELNNANAR
AMLARMLTIDPAERMSTEEALAHPYISLWFQEDEVNRQAPVPYDHALDEQELSLDQWKAL
LFQEIREIQAETLEAE

Cx. quinquefasciatus JNK CPIJ001157
MAYANFPLPELLNEINNNASNTEFTVPGRFQNLFPIGIGAQGAVCAAMDVVTGRPVAIKK

LSRPFQDVTHAKRAYREIKLMRLVDHPFIIKLLHAYSPQNSLDTFRDIYLFTERMDTNLS
VVIGNPLDHERLSFLVYQMLCGVKYLHSAGIIHRDLKPTNIVVRADCSLKILDFGLAKVV
GTNFMMTQYVVTRYYRAPEVILNMEYDTKVDIWAIGCIMAELITGRVLFPGTDHVDQWNK
IVETLGTPTPELIAKAPSSERRYIETLPVHPRPTIEQLFPDESFLATAAGSPELNNANAR
AMLARMLTIDPAERMSTEEALAHPYISLWFQEDEVNRQAPVPYDHALDEQELSLDQWKAL
LFQEIREIQAETLEAEAVESESESEPESVESGLFGDLESESESESSKLEQLESESEPESA

Orthologous *Ae. aegypti* and *Cx. quinquefasciatus* sequences for AGAP009461

Ae. aegypti JNK AAEL008634
FEVPSRYIRLEAKGFGAQGMVCAAYDTITQQNVAIKKLSRPFQNVTHAKRAYREFKLMKL
VNHKNIIGLLNAFTPQRTLEEFQDVYLVMELMDANLCQVIQMDLDHERMSYLLYQMLCGI
KHLHSAGIIHRDLKPSNIVVKSDCTLKILDFGLARTAGTTFMMTPYVVTRYYRAPEVILG
MGYKENVDIWSVGCIMGEMIRGGVLFPGTDHIDQWNKIIEQLGTPSPSFMTRLQPTVRNY
VENRPRYTGYPFDRLFPDVLFPTDSNEHNRLKASQARDLLSKMLVVDPEHRISVDQALVH
SYINVWYDESEVNAPAPGPYDHSVDEREHTVEQWKELIYQEVMEYEARNNLTDGEGTPR

Cx. quinquefasciatus JNK CPIJ001158
MSSSRHAFYTVEVGDTKFTILKRYQNLKPIGSGAQGIVCAAYDTITQQNVAIKKLSRPFQ
NVTHAKRAYREFKLMKLVNHKNIIGLLNAFTPQRTLEEFQDVYLVMELMDANLCQVIQMD
LDHERMSYLLYQMLCGIKHLHSAGIIHRDLKPSNIVVKSDCTLKILDFGLARTAGTTFMM
TPYVVTRYYRAPEVILGMGYKENVDIWSVGCIMGEMIRGGVLFPGTDHIDQWNKIIEQLG
TPSPSFMTRLQPTVRNYVENRPRYTGYPFDRLFPDVLFPTDSNEHNRLKASQARDLLSKM
LVVDPEHRISVDQALVHSYINVWYDESEVNAPAPGPYDHSVDEREHTVEQWKELIYQEVM
EYEARNNLADGDGTPR

Orthologous *Ae. aegypti* and *Cx. quinquefasciatus* sequences for AGAP012148

Ae. aegypti p38 MAPK AEAAEL008379
MPQFYKVEINKTEWEVPQKYQMLTPVGSGAYGQVCSATDTQHNVKVAIKKLARPFQSAVH
AKRTYRELRMLKHMNHENIIGLLDVFHPGGNTLDSFQQVYLVTHLMGADLNNIIRTQRLS
DDHVQFLVYQILRGLKYIHSAGIIHRDLKPSNIAVNEDCELKILDFGLARPTENEMTGYV
ATRWYRAPEIMLNWMHYNQTVDIWSVGCIMAELLTGRTLFPGTDHIDHLTRIMFFCGTPD
DELMQKITSEEARHYIRSLPKTEKRNFSDVFRGANPLAIDLLEKMLELDADKRVTAEQAL
AHPYLEKYADPTDEPTSSLYDQSFEDMDLPVEKWKELVFQEVLNFVPQQHAHIGSDGQ

Cx. quinquefasciatus p38 MAPK CPIJ002174
MVAPGRALVTNSATDSQHNAKVAIKKLARPFQSAVHAKRTYRELRMLKHMNHENIIGLLD
VFHPGGNTLDSFQQVYMVTHLMGADLNNIIRTQRLSDDHVQFLVYQILRGLKYIHSAGII
HRDLKPSNIAVNEDCELKILDFGLARPTENEMTGYVATRWYRAPEIMLNWMHYNQTVDIW
SVGCIMAELLTSRTLFPGTDPDLSITE

Cx. quinquefasciatus p38 MAPK CPIJ002175
MFFCGTPGDELMQKITSEEARHYIRSLPKTEKRNFSDVFRGANPLAIDLLEKMLELDADK
RITAEQALAHPYLEKYADPTDEPTSSLYDQSFEDMDLPVEKWKEVVNFVPQQHAHIGGDA
Q
