# Supplementary figures and images for: The mitogen-activated protein kinome from Anopheles gambiae: identification, phylogeny and functional characterization of the ERK, JNK and p38 MAP kinases
Source: BMC Genomics. 2011 Nov 23;12:574. doi: 10.1186/1471-2164-12-574 (PMC3233564; doi:10.1186/1471-2164-12-574)

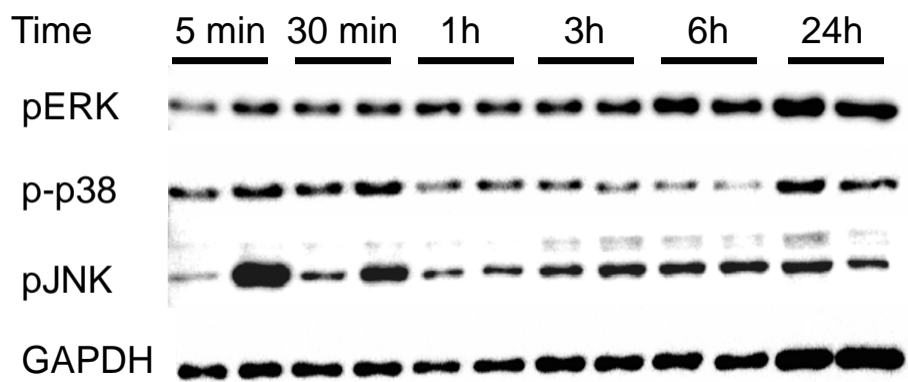

Supplement: Additional file 5 — LPS-induced MAPK phosphorylation in An. gambiae 4a3B cells. Cells were treated with 100 μg/ml LPS or an equivalent volume of PBS. Cells were collected at 5 min, 30 min, 1 h, 3 h, 6 h and 24 h after treatment. MAPK phosphorylation was examined by western blotting as described in the Methods. GAPDH levels provided an assessment of protein loading and were used to normalize corresponding phospho-MAPK levels. This figure is a representative of immunoblots from 6-9 independent experiments. [file 1471-2164-12-574-S5.PDF]

A

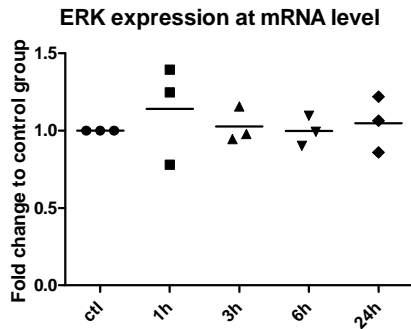

B

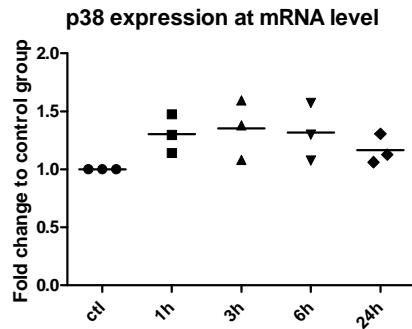

C

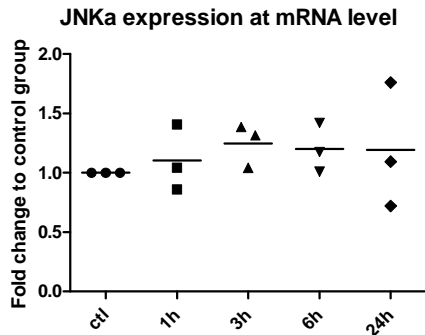

D

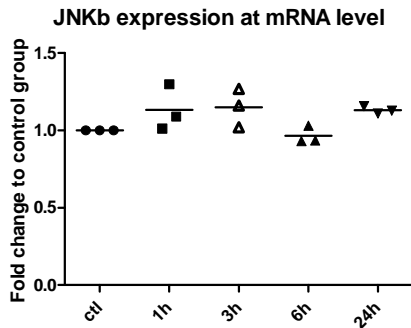

Supplement: Additional file 6 — MAPK transcript expression in insulin-stimulated An. gambiae 4a3B cells. Cells were treated with 1.7 μM human insulin or an equivalent volume of diluent and collected at 1 h, 3 h, 6 h and 24 h after treatment (n = 3 for control and treatment at each timepoint). Expression levels of ERK (A), p38 MAPK (B), JNKa (C), and JNKb (D) were analyzed by qPCR as described in the Methods. Expression of ribosomal protein S7, a housekeeping gene control, was used for normalization of treatment and control expression. Insulin-treated MAPK expression levels are shown as fold changes relative to the timepoint-matched control group levels. [file 1471-2164-12-574-S6.PDF]
